# Is the magnetic resonance imaging contrast agent gadoteric acid ototoxic?

**DOI:** 10.1016/j.bjorl.2026.101911

**Published:** 2026-09-22

**Authors:** Osman Cerlet, Kemal Keseroğlu, Unsal Han, Sibel Alicura Tokgöz, Melih Can Öner, Mehmet Burkay Karakulak, Guleser Saylam

**Affiliations:** a Ankara Bilkent City Hospital, Department of Otorhinolaryngology Head and Neck Surgery, Ankara, Turkey; b Private Practice, Clinic of Otorhinolaryngology, Ankara, Turkey; c Ankara Etlik City Hospital, Department of Pathology, Ankara, Turkey; d Ankara Etlik City Hospital, Department of Otorhinolaryngology Head and Neck Surgery, Ankara, Turkey; e Karaman Training and Research Hospital, Department of Otorhinolaryngology, Head and Neck Surgery, Karaman, Turkey; f University of Lokman Hekim Akay Hospital, Department of Otorhinolaryngology Head and Neck Surgery, Ankara, Turkey

**Keywords:** Auditory brainstem response, Ototoxicity, Gadoteric acid, Gadolinium-based contrast agent, MRI

## Abstract

**Objective:**

Contrast Magnetic Resonance Imaging (MRI) reveals endolymphatic hydrops in both symptomatic and asymptomatic ears. Radiological visualization of hydrops is important in the diagnosis of Meniere’s disease; however, the ototoxicity of intratympanically administered gadoteric acid remains unknown. To investigate the ototoxicity of the intratympanic administration of gadoteric acid at different doses using an Auditory Brainstem Response (ABR) test and histopathological methods.

**Methods:**

Forty healthy male Wistar Albino rats were randomly divided into four groups. The effect of different dilutions of gadoteric acid on the inner ear was examined using the ABR test at 10, 16, and 24 kHz. Basal measurements were compared with those at 1 and 14 days after injection. After completing the final measurements, the rats were sacrificed, and samples were taken for histopathological examination under light microscopy.

**Results:**

Following the intratympanic administration of gadoteric acid in rats, the ototoxicity was evaluated on days-1 and -14 based on ABR testing and histopathological examination. The ABR measurements demonstrated that pure gadoteric acid caused a significant decrease in the hearing threshold at 10, 16, and 24 kHz. By contrast, threshold shifts were not significant with diluted preparations. Histopathological examination revealed no significant differences.

**Conclusion:**

Ototoxicity was detected on day-14 following the intratympanic administration of undiluted gadoteric acid, determined by ABR testing. The dilution of gadoteric acid with saline at a 1:16 ratio appeared to be the safest dose.

*The level of evidence of this article is 5.* This article is an experimental animal and laboratory study.

## Highlights

• Intratympanic gadoteric acid may cause ototoxicity depending on concentration.
• Undiluted gadoteric acid caused significant ABR threshold shifts at 10–24 kHz.
• • Diluted gadoteric acid preparations did not demonstrate significant ototoxicity.
• Histopathology showed no significant structural damage in the inner ear.
• A 1:16 saline dilution of gadoteric acid is the safest intratympanic dose.

## Introduction

Meniere’s disease is an inner ear disorder that affects both balance and hearing. Its underlying pathology is Endolymphatic Hydrops (EH), first identified through autopsy in patients with Meniere’s disease.^1^ More recently, the visualization of EH using Magnetic Resonance Imaging (MRI) has led to advancements in diagnosis. A significant proportion of contrast materials used in MRI examinations contain gadolinium. In EH imaging, the contrast material can be administered intravenously or intratympanically before the procedure.^2^ Images obtained using intratympanic contrast materials have gained rapid acceptance owing to their high quality.^3^ In an animal model experiment, Zou et al.^4^ demonstrated the passage of contrast material containing gadodiamide into the inner ear after injection. As a result, MRI examinations performed with contrast material administered intravenously or intratympanically started to play an important role in the diagnosis of EH and Meniere’s disease.^5^ In addition to helping establish the diagnosis of Meniere’s disease, it also plays a significant role in disease follow-up.^6^, ^7^, ^8^ Gadolinium-containing contrast materials have known systemic side effects, such as nausea, vomiting, headache, and contrast nephropathy.^9^ However, the ototoxicity of these contrast materials has not yet been clarified.

Tanaka et al.^10^ demonstrated that gadolinium hydrate is ototoxic in frogs, while gadoteridol has been reported to cause mild cytotoxicity in cochlear outer hair cells.^11^ Nonoyama et al.^12^ compared the ototoxicity of gadoteridol and gadodiamide in rats, and although no gadoteridol toxicity was observed, gadodiamide was shown to be ototoxic. In human trials, no effect on hearing in the short- and long-term has been observed following the intratympanic administration of diluted gadodiamide and gadopentetate meglumine^13^^,^^14^ to the best of our knowledge; no previous studies have investigated the ototoxicity of gadoteric acid.

Imaging of hydrops after the intratympanic application of contrast material is important for the diagnosis of Meniere’s disease; thus, this study aimed to investigate the ototoxicity of intratympanically administered gadoteric acid in rats using electrophysiological and histopathological evaluations.

## Methods

All animal experiments were approved by the Local Ethical Committee for Animal Research. The experimental protocol of the study complied with the ‘Reporting Data on Auditory Brainstem Responses (ABR) in Rats: Recommendations Based on Review of Experimental Protocols and Literature ‒ 2021’ guidelines.^15^

### Experiment animals

The study sample comprised 44 healthy male 12-month-old Wistar albino rats, each weighing 250–300 g. The rats were housed in steel cages under standard laboratory conditions ‒ including a 12-h light/dark cycle, a temperature of 24°C ± 2 °C, and 35%–60% humidity ‒ with unrestricted access to fresh food and drinking water.

A randomized experimental procedure was used in this study. Before selecting the experimental rats, the external ear canal and tympanic membranes were first examined with an otomicroscope (Zeiss, Germany) after subcutaneous anesthesia was administered with 90 mg/kg ketamine (Ketalar flacon; Pfizer Drugs, Istanbul) and 3 mg/kg xylazine (Rompun flacon; Bayer, Istanbul, Turkey). In rats with no pathology in the external ear or tympanic membrane, basal hearing thresholds were determined using the ABR test with an Intelligent Hearing Systems device (IHS, Miami, FL, USA).

### Formation of the groups

The study initially included 44 rats divided into four groups of 11 rats each; the right ear of each rat was used for the analysis. During the study, one rat in groups A and B, and two rats in groups C and D died. These animals were not replaced, and final evaluations were performed on a total of 38 rats.

• Group A (n = 10): Control group, received a single intratympanic administration of 0.05 mL physiological saline.
• Group B (n = 10): Received a single intratympanic administration of 0.05 mL gadoteric acid (Dotarem, 0.5 mmoL/mL; Guerbet, Istanbul).
• Group C (n = 9): Received a single intratympanic administration of 0.05 mL gadoteric acid (Dotarem, 0.5 mmoL/mL; Guerbet, Istanbul) diluted 1:8.
• Group D (n = 9): Received a single intratympanic administration of 0.05 mL gadoteric acid (Dotarem, 0.5 mmoL/mL; Guerbet, Istanbul) diluted 1:16.

The baseline threshold levels for each rat were determined during the initial measurements. Subsequently, the drugs specific to each experimental group were administered intratympanically into the middle ear under anesthesia with the assistance of an otomicroscope. Following the procedure, the animals were positioned so that the treated ear remained uppermost, and this position was maintained for 30-minutes to ensure adequate distribution of the medication.

On the 14th day after drug administration, one rat each from groups C and D died before euthanasia and final measurements. Histopathological evaluations were performed; however, their ABR results were not included in the analysis.

### Hearing evaluation and anesthesia administration

Rats that underwent initial hearing evaluations via ABR testing also underwent intratympanic contrast material application. Before the ABR measurements following intratympanic injection, all treated tympanic membranes were re-evaluated under otomicroscopy, and no pathology was observed. Hearing was reevaluated via ABR testing on days 1 and 14. After the final measurements, the rats were euthanized, the cadavers were decapitated, the temporal bone was dissected, and the cochlea was removed. The cochleae were fixed in formaldehyde for histopathological examination.

### Auditory brainstem response

A probe appropriate for the external ear canals of the rats was attached to E-A-RTone 3A headphone tubes for low-frequency measurements, and high-frequency transducers for high-frequency ABR measurements. Negative needle electrodes were placed on the mastoid of the tested ear, positive electrodes on the forehead, and earth electrodes on the side of the contralateral foot.

Electrode impedance levels were carefully checked and maintained within the range of 0–3 kΩ. Stimuli were provided in the form of a stimulus tone burst (10, 16, and 24 kHz). The repetition rate was set at 21.1 s, the time window was 15 ms, and 2000 samples were obtained for signal averaging. Frequencies of 100 Hz and 1500 Hz were used for high-pass and low-pass filtering, respectively. The hearing threshold was determined using the descending-ascending method, starting at 70 dB. The hearing threshold was set at the lowest level that could be determined and repeated. ABR thresholds II‒III were defined as the lowest severity level of the wave complex observed in each measurement for each rat.

The threshold values of the first measurements for each rat were defined; under anesthesia, the drugs were then intratympanically administered to the middle ear of the rats under otomicroscopic guidance. The second and third ABR measurements were performed on days 1 and 14 after drug administration, and the threshold hearing values for each rat were determined based on these measurements. The results were statistically compared within groups with respect to the change between days and frequency-specific changes.

### Histopathological examination

The excised temporal bone materials were fixed in 10% formaldehyde; after 24 h, they were placed in a 10% formic acid solution for 2-days for decalcification. The solution was changed daily. After 48 h, the samples were split into two parts parallel to the outer ear pathway by a pathologist blinded to the groups. The samples were washed under running tap water, and routine paraffin follow-up was performed for 18 h using a Leica SP300 device. The samples were stained with hematoxylin and eosin and evaluated under a binocular light microscope. Sample sections of the cochlea were evaluated with respect to changes observed in the organs of Corti, stria vascularis, and spiral ganglion.

Hair cells in the organ of Corti were scored subjectively as 0 = no damage, 1 = mild damage, 2 = moderate damage, and 3 = severe damage, considering the cell integrity, arrangement, and characteristics of the nucleus and cytoplasm. Changes in the stria vascularis were scored as 0 = none, 1 = mild, 2 = moderate, and 3 = severe, considering edema, cytoplasmic vacuolization, and intermediate cell atrophy. Spiral ganglion changes were scored in the same categories as the stria vascularis according to vacuolization and nuclear damage.^16^

### Statistical analysis

Power analysis was performed using the G*Power software. To detect a large level of difference (effect size: w = 0.5) among the four groups with respect to positivity in the immunohistochemical examination, it was necessary to have a total of 44 rats (11 in each group) to provide a power of 80% and 5% type 1 error.

ABR measurements and histopathological variables were compared among the four groups. The data obtained in this study were statistically analyzed using SPSS 23 (IBM, USA) software. Numerical variables were reported as mean ± Standard Deviation (SD) or median (25th–75th percentage) values, and categorical variables as number (n) and percentage (%). Friedman two-way analysis of variance was used for within-group comparisons. Differences were considered statistically significant at p < 0.05.

## Results

### Intragroup evaluations of the ABR results

#### 10 kHz hearing threshold results

The 10 kHz ABR threshold measurement results for all groups are presented in Table 1.

• Group A: No significant changes were observed between the basal, 1st and 14th day threshold values (p > 0.05).
• Group B: A significant increase was observed between the basal, 1 st, and 14th day threshold values (p = 0.005). The increase in threshold value between the baseline and 14th day measurements was statistically significant (p = 0.022). The mean threshold values were 34 ± 8.43 dB at baseline, and 47 ± 10.59 dB on the 14th-day.
• Group C: No significant changes were observed between the basal, 1st and 14th day threshold values (p > 0.05).
• Group D: No significant changes were observed between the basal, 1st and 14th day threshold values (p > 0.05).

**Table 1:** 10 kHz threshold values and p-values of each group.

Group	Threshold values (dB HL)				
	Frequency	Day	Mean	SD	p-Value
A	10 kHz	0	37.00	9.487	>0.05
		1	36.00	10.750	
		14	41.00	13.703	
B	10 kHz	0	34.00	8.433	0.005^a^
		1	41.50	8.835	
		14	47.00	10.593	
C	10 kHz	0	32.22	4.410	>0.05
		1	35.00	7.906	
		14	41.11	15.161	
D	10 kHz	0	30.56	8.079	>0.05
		1	33.33	5.000	
		14	36.11	7.817	

a A significant difference was observed between the threshold values on days 0 (34 dB) to 14 (47 dB).

#### 16 kHz hearing threshold results

The 16 kHz ABR threshold measurement results for all groups are presented in Table 2.

• Group A: No significant changes were observed between the basal, 1st and 14th day threshold values (p > 0.05).
• Group B: A significant increase was observed between the basal, 1st and 14th day threshold values (p = 0.002). The increase in threshold values between the baseline measurement and the 1st day, and between the baseline measurement and the 14th day were determined to be significant (Basal – 1st-day: p = 0.04; Basal – 14th-day: p = 0.004). The mean threshold values were 39 ± 9.94 dB at baseline, 49.5 ± 7.97 dB on the 1st-day, and 51.1 ± 12.44 dB on the 14th-day.
• Group C: No significant change was observed between the basal, 1st and 14th day threshold values (p > 0.05). Although the mean threshold values increased each day, no significant increase was observed within the group.
• Group D: No significant change was observed between the basal, 1st and 14th day threshold values (p > 0.05). Although the mean threshold values increased each day, no significant increase was observed within the group.

**Table 2:** 16 kHz threshold values and p-values of each group.

Group	Threshold values (dB HL)				
	Frequency	Day	Mean	SD	p-Value
A	16 kHz	0	42.50	10.35	>0.05
		1	40.00	12.247	
		14	43.33	10.000	
B	16 kHz	0	39.00	9.944	0.002^a^
		1	49.50	7.976	
		14	51.11	12.444	
C	16 kHz	0	40.00	14.142	>0.05
		1	40.71	17.895	
		14	49.44	16.287	
D	16 kHz	0	30.00	10.690	>0.05
		1	41.11	13.642	
		14	45.00	12.247	

a A significant difference was observed between the threshold values on days 0 (39 dB) to 1 (49.50 dB), and 0 (39 dB) to 14 (51.11 dB).

#### 24 kHz hearing threshold results

The 24 kHz ABR threshold measurement results for all groups are presented in Table 3.

• Group A: No significant changes were observed between the basal, 1st and 14th day threshold values (p > 0.05).
• Group B: A significant increase was observed between the basal, 1st and 14th day threshold values (p = 0.002). The increase in the threshold value between the baseline and 14th day measurements was determined to be significant (baseline –14th day: p = 0.004). The mean threshold values were 35 ± 13.54 dB at baseline, 40.5 ± 12.79 dB on the 1st day, and 51.67 ± 13.22 dB on the 14th day. Although an increase was observed in the mean threshold value on day-1 compared to baseline, this increase was not significant.
• Group C: No significant change was observed between the basal, 1st and 14th day threshold values (p > 0.05). Although the mean threshold values increased each day, this increase was not significant.
• Group D: No significant change was observed between the basal, 1st and 14th day threshold values (p > 0.05). Although the mean threshold values increased each day, this increase was not significant.

**Table 3:** 24 kHz threshold values and p-values of each group.

Group	Threshold values (dB HL)				
	Frequency	Day	Mean	SD	p-Value
A	24 kHz	0	42.50	10.351	>0.05
		1	40.00	12.247	
		14	42.50	10.351	
B	24 kHz	0	35.00	13.540	0.002^a^
		1	40.50	12.791	
		14	51.67	13.229	
C	24 kHz	0	27.00	11.877	>0.05
		1	34.38	11.783	
		14	35.56	10.138	
D	24 kHz	0	28.33	12.748	>0.05
		1	32.22	19.221	
		14	38.33	18.708	

a A significant difference was observed between the threshold values on days 0 (35 dB) to 14 (51.67 dB).

### Histopathological evaluation results

Changes in the stria vascularis and spiral ganglion were scored in both groups. The histopathological evaluation results for the hair cells of the organs of Corti, stria vascularis, and spiral ganglion are shown in Table 4.

**Table 4:** Evaluation of damage and p-values of each group in the histopathological examinations.

Group	Organ of corti evaluation				Stria vascularis and spiral ganglion evaluation			
	None (0)	Mild (1)	Moderate (2)	Severe (3)	None (0)	Mild (1)	Moderate (2)	Severe (3)
A (n = 10)	10 (100%)	0	0	0	10 (100%)	0	0	0
B (n = 10)	8 (80%)	2 (20%)	0	0	9 (90%)	1 (10%)	0	0
C (n = 10)	9 (90%)	1 (10%)	0	0	10 (100%)	0	0	0
D (n = 10)	10 (100%)	0	0	0	10 (100%)	0	0	0
p = 0.187				p = 0.415				

Histopathological evaluation in Group B revealed mild morphological damage in the organ of Corti in two rats, and in the stria vascularis and spiral ganglion in one rat. In group C, mild morphological damage was observed in the organ of Corti in one rat. No morphological changes were observed in any of the rats in groups A or D (Fig. 1, Fig. 2). The damage was not histopathologically significant (Table 4).

**Fig. 1:**
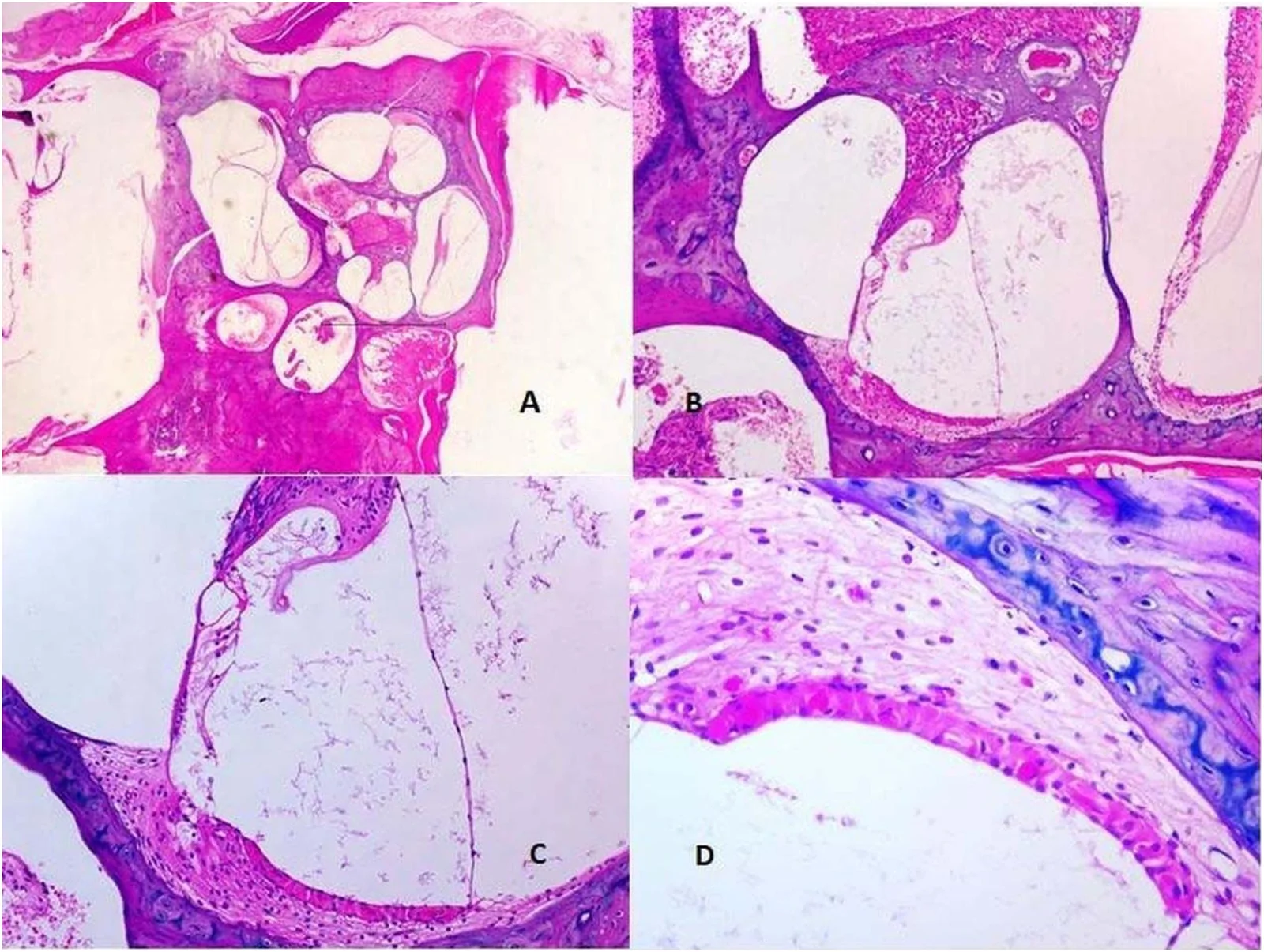
(a) Group A (control group) cochlea vertical slice showing normal morphology (H&E, ×4), (b) Group A unit with 3 foci (Scala tympani, Scala vestibulum and cochlear ductus) (H&E, ×10), (c) Group A organ of Corti containing support cells with regularly organized hair cells (H&E, ×40), (d) Group A stria vascularis showing normal morphology (H&E, ×40).

**Fig. 2:**
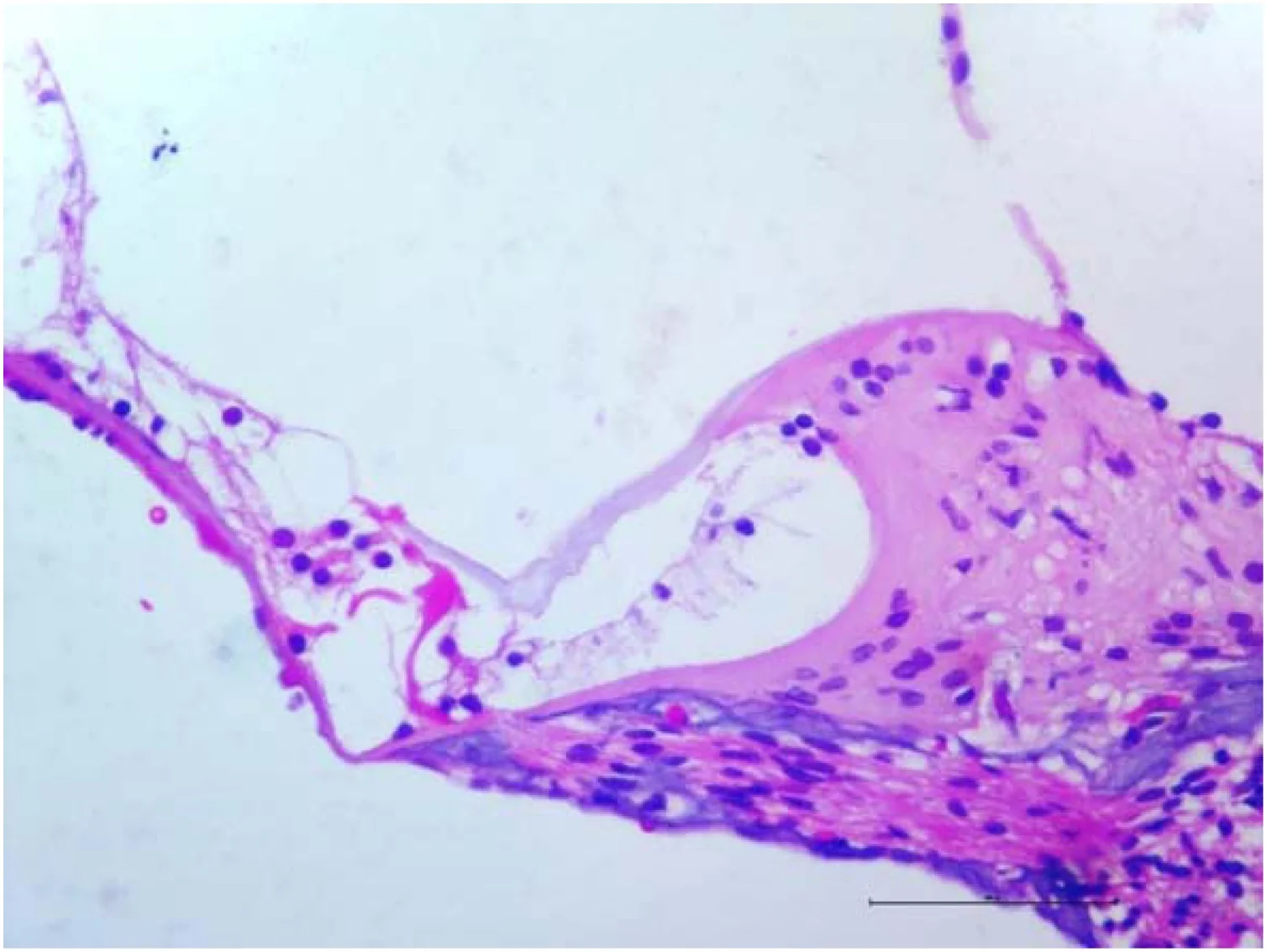
Group B – light microscope appearance of mild damage in the hair cells of the organ of Corti (H&E, ×100).

## Discussion

With advances in technology, imaging methods have played an important role in the diagnosis of many diseases, including Meniere’s disease. The use of contrast materials containing gadolinium prior to imaging has contributed to the identification of various pathologies. Intratympanic, rather than intravenous administration allows these contrast materials to pass through the round window to the inner ear. Thus, imaging can be performed with a smaller amount of contrast material, thereby lowering the risk of systemic side effects related to the contrast agents. This experimental animal study aimed to investigate the ototoxicity of gadoteric acid, which can be intratympanically administered during MRI, thereby contributing to the diagnosis of Meniere’s disease.

In this study, the results of the electrophysiological ABR test at all frequencies demonstrated the ototoxicity of pure gadoteric acid. In addition to the ototoxicity determined at all frequencies of the ABR results on day-14, ototoxicity was also observed in the 16 kHz measurements on day-1. In histopathological examinations of the inner ear structures, this ototoxicity was not significant; however, when evaluated together with the electrophysiological results, undiluted gadoteric acid was determined to be ototoxic. In a study by Kakigi et al.,^17^ morphological changes were evident in the stria vascularis and organs of Corti after the application of pure gadodiamide to the inner ear; this pure concentration was found to be ototoxic. Physiological and morphological changes in the stria vascularis and organs of Corti cause toxicity to the ABR threshold values. The toxicity of a high concentration of gadoteric acid at the cellular level suggests that this toxicity is similar to the dose-dependent toxicity of gadodiamide,^18^ which was clearly observed when compared to the diluted concentrations in the current study.

The concentrations of the test solutions were determined based on their ability to examine EH after intratympanic gadodiamide injection, and were diluted at 1/8 or 1/16 ratios, as described by Nakashima et al.^19^ Following their study, the diluted forms were found to be sufficient for MRI, and contrast material containing a higher concentration of gadolinium was not required. Similarly, in the current study, gadoteric acid at 1/8 and 1/16 diluted concentrations was administered intratympanically, and no ototoxicity was observed in the inner ear. The electrophysiological test results were similar to those of the control group administered physiological saline.

In an experimental animal study using frogs, Tanaka et al.^10^ showed that the 1/4 diluted form of gadolinium hydrate caused morphological deterioration in the vestibular end organs; however, gadolinium hydrate diluted by 1/8 and 1/16 caused less deterioration. The absence of histopathological ototoxicity, even in the group wherein pure gadoteric acid was used, raises the question of whether it is safer than gadolinium hydrate. To answer this question, further studies comparing the ototoxicity of gadodiamide and gadoteric acid are required. Louza et al.^13^^,^^14^ reported that gadodiamide and gadopentetate dimeglumine demonstrated no short-term ototoxicity or long-term toxicity, respectively, in humans. However, to date, no studies have investigated the ototoxicity of intratympanically administered gadoteric acid. Following the intratympanic administration of gadoteric acid to rats in this study, ototoxicity was investigated, and the question of whether there was a difference between doses with respect to ototoxic findings was raised. This study was hence designed to mimic the application of contrast materials other than gadoteric acid. The most important aspect of the current study is that it is the first experimental animal study to electrophysiologically and histopathologically investigate the ototoxicity of GA. However, there were some limitations; the systemic passage of intratympanically administered gadoteric acid was not examined; thus, toxicity due to systemic passage was not evaluated, including the lack of comparative studies with other gadolinium-containing contrast agents, the short follow-up period, and the small sample size. The ratio of diluted forms that can pass through the inner ear remains unknown; therefore, further studies are needed to determine the optimal passage for MRI. Still, this study paves the way for further MRI studies to be conducted using different dilutions.

## Conclusion

The results of this study demonstrate that pure gadoteric acid administered intratympanically is ototoxic in the ABR test after 14-days. Gadoteric acid diluted with saline at a 1/16 ratio was found to be the safest form; however, the fact that no ototoxicity was observed with the 1/8 diluted form revealed that this dosage is safe. A mismatch between functional and structural damage was observed, and this discrepancy may be clarified through studies with longer follow-up periods.

## ORCID IDs

Kemal Keseroğlu: 0000-0002-6589-1663

Unsal Han: 0000-0003-1952-4499

Sibel Alicura Tokgöz: 0000-0002-5058-1400

Melih Can Öner: 0000-0002-7810-0461

Mehmet Burkay Karakulak: 0009-0003-4928-6986

Guleser Saylam: 0000-0002-6499-7140

## Funding

None.

## Data availability

The data supporting the findings of this study are available from the corresponding author upon reasonable request.

## CRediT authorship contribution statement

**Osman Cerlet:** Conceptualization, Methodology, Writing – original draft, Writing – review & editing. **Kemal Keseroğlu:** Supervision, Writing – review & editing. **Unsal Han:** Data curation, Formal analysis. **Sibel Alicura Tokgöz:** Validation, Visualization. **Melih Can Öner:** Software. **Mehmet Burkay Karakulak:** Data curation, Formal analysis. **Guleser Saylam:** Project administration.

## Declaration of competing interest

The authors declare no conflicts of interest.
